# Is Social Gender Transition Associated with Mental Health Status in Children and Adolescents with Gender Dysphoria?

**DOI:** 10.1007/s10508-023-02588-5

**Published:** 2023-04-04

**Authors:** James S. Morandini, Aidan Kelly, Nastasja M. de Graaf, Pia Malouf, Evan Guerin, Ilan Dar-Nimrod, Polly Carmichael

**Affiliations:** 1 School of Psychology, The University of Sydney, Sydney, NSW 2006 Australia; 2 Gender Service Organization, Kelly Psychology, London, UK; 3 Center of Expertise on Gender Dyphoria, VU University Medical Center, Amsterdam, The Netherlands; 4 King Street Psychology Clinic, Newtown, Australia; 5 School of Behavioural and Health Sciences, Australian Catholic University, Sydney, Australia

**Keywords:** Gender dysphoria, Social gender transition, Depression, Anxiety, Pediatric

## Abstract

Social gender transition is an increasingly accepted intervention for gender variant children and adolescents. To date, there is scant literature comparing the mental health of children and adolescents diagnosed with gender dysphoria who have socially transitioned versus those who are still living in their birth-assigned gender. We examined the mental health of children and adolescents referred to the Gender Identity Development Service (GIDS), a specialist clinic in London, UK, who had socially transitioned (i.e., were living in their affirmed gender and/or had changed their name) versus those who had not socially transitioned. Referrals to the GIDS were aged 4–17 years. We assessed mental health correlates of living in one’s affirmed gender among 288 children and adolescents (208 birth-assigned female; 210 socially transitioned) and of name change in 357 children and adolescents (253 birth-assigned female; 214 name change). The presence or absence of mood and anxiety difficulties and past suicide attempts were clinician rated. Living in role and name change were more prevalent in birth-assigned females versus birth-assigned males. Overall, there were no significant effects of social transition or name change on mental health status. These findings identify the need for more research to understand how social transition influences mental health, including longitudinal studies that allow for more confident inferences to be made regarding the relationship between social transition and mental health in young people with gender dysphoria.

**Supplementary Information:**

The online version contains supplementary material available at 10.1007/s10508-023-02588-5.

## Introduction

There are many unanswered questions regarding how best to support children and adolescents who experience gender dysphoria, a term used to describe persistent distress related to one’s biological sex/sex characteristics and/or birth-assigned gender (Zucker et al., 2016). One such question is—at what age and under what conditions do children and adolescents who experience gender dysphoria benefit from social gender transition (i.e., living in their affirmed gender rather than their gender assigned at birth, which may involve changing their physical gender markers such as hair and clothing as well as their name and gender pronouns)?^1^ Answering this question is of particular urgency, given that children and adolescents who experience gender dysphoria or are otherwise gender variant demonstrate a higher prevalence of mental health difficulties than their cisgender peers (Becerra-Culqui et al., 2018). A recent systematic review of psychiatric comorbidities among prepubertal children diagnosed with gender dysphoria (aged 12 and under) (Frew et al., 2021) demonstrated that up to 21% met criteria for an anxiety disorder, almost half had a significant psychiatric history, and around 10% had attempted suicide. A systematic review of adolescents experiencing gender dysphoria (aged 12–18) (Thompson et al., 2022) demonstrated that comorbid mental health issues were present in 22–78%. Specifically, the prevalence of mood disorders ranged from 30 to 78%, anxiety disorders from 21 to 63%, and suicidal ideation from 12 to 74%. Given that social gender transition is theorized to ameliorate mental health issues in gender dysphoric young people (Ehrensaft, 2016), further empirical examination of this intervention is warranted.

Before moving forward, it is important to clarify terminology. Young people who experience gender dysphoria are variously referred to as “gender variant” (Riley et al., 2011; Wong et al., 2019), “transgender” (Durwood et al., 2017; Olson et al., 2016), “cross-gender identified” (Kuvalanka et al., 2017), and “gender dysphoric” (Wallien & Cohen-Kettenis, 2008)—although these respective terms can imply important sample differences in some instances.^2^ In the present study, we use the term gender variant to capture the diversity of presentations (and stages of gender identity development and symptom severity) among children and adolescents referred to specialist gender dysphoria services.

Disagreement over when social gender transition is indicated is what most differentiates competing models of care for gender dysphoric young people (de Vries & Cohen-Kettenis, 2012; Ehrensaft et al., 2018; Steensma & Cohen-Kettenis, 2018; Zucker, 2018). Perhaps the most widely endorsed model of care, the affirmative model, takes a non-pathologizing stance toward gender variance in young people and views a “myriad of healthy [non-cisgender] gender outcomes” as possible (Ehrensaft, 2016). This approach prioritizes following the child’s lead and accepting and facilitating expression of the child’s “true-gendered-self” (i.e., the child’s authentic gender identity and expression) (Ehrensaft, 2016). A guiding concern underpinning this approach is the need to protect the child from harm associated with being raised in the wrong gender. Indeed, cultural and familial pressures to conform to cisgender identities, gender roles, and expression are thought to cause considerable harm as they require a gender variant child to suppress their authentic self and emulate socially expected gender roles (the “false gendered self”). Ehrensaft et al. (2018) contend that the construction of a false gendered self is a key contributor of psychiatric morbidity, including suicidality, observed in gender variant populations.

Others have suggested that clinicians and parents should, where possible, delay social gender transition (Steensma et al., 2013). This is based on evidence that gender dysphoria (and cross-gender identities) frequently desist in prepubertal children (Drummond et al., 2008; Singh et al., 2021; Zucker, 2018) and that premature social gender transition may foreclose the child’s gender identity development, increasing the likelihood that gender dysphoria will persist (possibly necessitating medical transition in adolescence onward). This approach has been referred to as “watchful waiting” (de Vries & Cohen-Kettenis, 2012).

There are an increasing number of prepubertal children pursuing social transition prior to attending specialist gender services. For example, the Amsterdam Gender Identity Clinic reported that, before the year 2000, 1.7% of children who attended the clinic were completely socially transitioned at first presentation, while between 2000 and 2004, 3.3% had completed social transitioning at first presentation (Steensma & Cohen-Kettenis, 2011). Reflecting a more dramatic and recent shift, the proportion of birth-assigned males who had socially transitioned prior to contact with the Tavistock Gender Identity Development Service in London, increased from 19.8% in 2012 to 47.2% in 2015 (Morandini et al., 2022). This shift toward social transition being more common prior to contact with gender services may reflect increasing cultural acceptance of transgender identities as viable and healthy outcomes for children and adolescents (Brunskell-Evans & Moore, 2019; Ehrensaft, 2016) and, therefore, greater comfort of parents in independently facilitating a child’s social gender transition.

Existing research on the mental health correlates of social gender transition utilizes diverse methodologies and focuses on somewhat distinct populations. Tracking this research chronologically—the first notable study was Kuvalanka et al.’s (2017) qualitative study of five parents of transgender girls (birth-assigned males) between the ages of 8 and 11. Kuvalanka et al. found that according to parents, social transition appeared to reduce distress and increase self-esteem and self-confidence among their children. Other reports from clinicians and parents following early childhood social transition have echoed similar findings, reporting improved mood in children and enhanced peer and caregiver relationships (Wong & Drake, 2017), as well as being viewed as protective for the child’s happiness and well-being (Horton, 2022).

Several quantitative studies now exist examining correlates of social gender transition. Kuvalanka et al. (2017) examined the well-being of 45 children (aged 6–12 years) in the community (volunteered by parents) who were supported in their gender identity. Kuvalanka et al. compared children with “cross-gender identities” (i.e., those that identified as trans girls or trans boys) and those who were gender nonconforming or had uncertain gender identities (labeled as having “non-cross-gender identities”) to normed data on the Child Behavior Checklist (CBCL). Cross-gender identified children demonstrated functioning in the normal range on all three measured indices (internalizing problems, externalizing problems, and total problems). On the other hand, those with non-cross-gender-identities were in the borderline clinical or clinical range on the same indices. When the two groups of gender diverse children were compared, those who were cross-gender identified demonstrated superior outcomes on internalizing problems and total problems, suggesting binary transition may be protective against mental health difficulties in gender diverse populations—and that socially transitioned children can demonstrate psychological well-being comparable to cisgender controls.

The most widely cited quantitative studies assessing mental health in social gender transitioned are those by Durwood et al. (2017) and Olson et al. (2016). These studies, using the Patient Reported Outcomes Measurement Information System (PROMIS), compared mental health among community convenience samples of American and Canadian prepubescent children who had fully socially transitioned with cisgender siblings and matched controls. The first study (Olson et al., 2016), which was based on parent reports of 73 (51 birth-assigned males) socially transitioned transgender children (3- to 12-years-old), found levels of depression and anxiety in this group was largely comparable with matched controls and siblings/peers, although trans-children were found to have slightly elevated rates of anxiety compared with national population averages. Additionally, Olson et al. compared their study’s findings (of socially transitioned children) to previous clinical samples of children reporting gender dysphoria, which included transgender children and those that had not yet transitioned or that may have identified as non-binary. Olson et al. found lower internalizing of symptoms in their sample of socially transitioned children, concluding that social transitioning may reduce mental health difficulties in gender variant youngsters.

The second study was based on parent and self-report reports of 116 socially transitioned transgender young people (68 birth-assigned males) (Durwood et al., 2017). This study found that among 9- to 14-year-old transgender young people, depression did not differ from matched-control or sibling peers, but that transgender young people, again, demonstrated slightly elevated anxiety. Additionally, among 6- to 14-year-old transgender young people, self-worth did not differ from cisgender matched controls or cisgender siblings. Collectively, these findings have been interpreted to suggest that affirming a gender variant child/adolescents’ gender identity via social transition will reduce psychological difficulties often observed within gender variant populations (de Graaf et al., 2018; de Vries et al., 2016; Ehrensaft et al., 2018). The Olson et al. (2016), Durwood et al. (2017), and Kuvalanka et al. (2017) studies suggest that social transition can be associated with normative mental health outcomes among young people with gender dysphoria, a group who have been shown to experience poorer psychological well-being on the whole (Tankersley et al., 2021; Thompson et al., 2022).

Other recent studies, however, have failed to find superior well-being in socially transitioned young people. Wong et al. (2019) compared published CBCL data (van der Miesen et al., 2018) on 162 cisgender children (aged 6–12 years) who had levels of gender variance similar to children referred to specialist gender clinics, with published data on 104 children who had undertaken social gender transition (Kuvalanka et al., 2017; Olson et al., 2016). A statistical bootstrapping approach was utilized to control for birth-assigned sex, age, and degree of gender variance when comparing CBCL scores between cisgender gender variant children and socially transitioned gender variant children. Cisgender gender variant children and socially transitioned children demonstrated broadly equivalent levels of internalizing problems—and only a minority of each sample demonstrated clinical or borderline clinical scores on internalizing problems. This latter finding suggests that Olson et al.’s (2016) finding of broadly comparable mental health status between social gender transitioned children and normative samples might not be entirely surprising.

Finally, a study by Sievert et al. (2021) more directly examined whether social gender transition was related to improved psychological functioning in 54 gender variant children who had received a gender dysphoria diagnosis (aged 5– to 11-years). Social transition was assessed in a graded manner from 1 (no social transition and living in birth-assigned gender) to 4 (complete social transition in all life areas). After controlling for gender assigned at birth, age, socioeconomic status, poor peer relations, and general family functioning, social transition status did not predict psychological functioning as measured by the CBCL.

The existing literature has shown mixed evidence for a relationship between social gender transition and psychological functioning (positive effects in some studies and null effects in others). It should be noted, however, that the existing literature is limited in a number of respects. Past studies have failed to examine how the mental health consequences of social transition may be moderated by a range of individual factors, such as birth-assigned gender and pre- versus post-pubertal age. This may be partly due to the relatively small samples (*N*’s = 45–162) utilized in past studies, which precluded such analyses. Except for Durwood et al. (2017) (who included 6- to 14-year-olds), existing studies have been conducted in children aged 12 years of age or under. Given the recent preponderance of gender dysphoria first becoming apparent in adolescence (Aitken et al., 2015; de Graaf et al., 2018), examining the mental health correlates of social gender transition in early, mid, and late adolescence is increasingly clinically important as well. Next, past studies examining correlates of social transition have utilized self-report and parent-report measures (typically the CBCL) in assessing mental health. No studies to date have included ratings of mental health status by trained mental health professionals. Apart from Sievert et al. (2021), existing studies have not compared the mental health outcomes of children and adolescents diagnosed with gender dysphoria based on their social transition status, i.e., comparing socially transitioned versus non-socially transitioned gender dysphoric children and adolescents in terms of their mental health. Existing studies have compared transgender participants with their cisgender siblings or with normative data based on cisgender populations (Durwood et al., 2017; Olson et al., 2016), with gender variant cisgender individuals (Wong et al., 2019) or with non-binary children (Kuvalanka et al., 2017). Given that some commentators contend that transgender children and adolescents differ in kind to their gender variant cisgender peers (Temple Newhook et al., 2018), comparing correlates of social gender transition status among a population of children and adolescents all diagnosed with gender dysphoria provides a more direct test of proposed benefits of social gender transition. Failing to ensure that both socially transitioned and non-socially transitioned referrals to a gender service were nevertheless still experiencing gender dysphoria could possibly confound findings. For example, patients living in their birth-assigned gender may be found to have superior well-being because they were less likely to be gender dysphoric to begin with and, therefore, less distressed (not because avoidance of social transition leads to superior outcomes).

### The Present Study

There are scarce data comparing the mental health of gender dysphoric children and adolescents who have socially transitioned with those who have not (and who are living in their gender assigned at birth). The present study sought to contribute to this literature by undertaking this comparison in a cohort of children and adolescents who had presented for assessment at a specialist gender identity clinic in the UK. We aimed to extend on past studies in a number of ways, including: (1) utilizing a larger sample of socially transitioned children and adolescents; (2) examining whether associations between mental health and social transition were moderated by birth-assigned gender and developmental stage (by including patients from early childhood through to late adolescence); and (3) utilizing clinician ratings of mental health based on a comprehensive mental health assessment—potentially reducing risk of social desirability bias and complementing past studies of this type that have relied exclusively on parent or self-report data. Our study examines correlates of social gender transition on mood and anxiety disorders, given these disorders appear to be prevalent psychiatric comorbidities among gender variant youth (Frew et al., 2021; Thompson et al., 2022). We also examined the link between social gender transition and suicide attempts, given increased prevalence of this behavior in gender variant versus cisgender young people (Biggs, 2022), and the theorized link between transition status and suicidality (Ehrensaft et al., 2018).

## Method

### Subjects

**Fig. 1:**
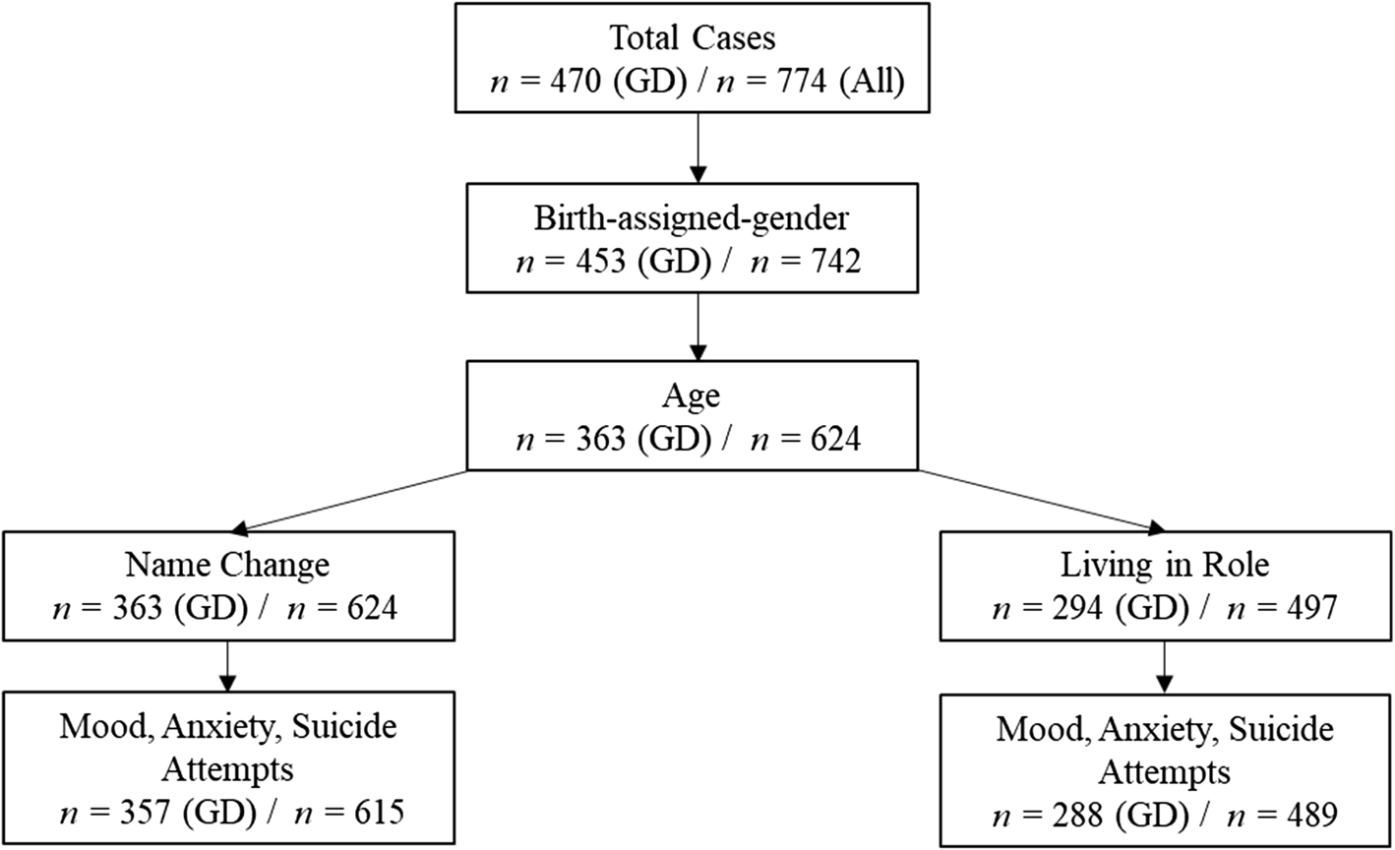
Flowchart outlining progressive exclusion of cases due to missing data and depicting final sample size for regression models examining name change status on mood, anxiety, and suicide attempt, and living in role status on mood, anxiety, and suicide attempt. Left-hand sample size reflects cases where a positive gender dysphoria (GD) was recorded (primary analyses reported in the manuscript were conducted upon this sample). Right hand size represents total cases, inclusive of patients with a positive gender dysphoria diagnosis recorded (*n* = 470), a negative gender dysphoria diagnosis recorded (*n* = 155), or for which information on gender dysphoria status was absent (*n* = 149). Analyses reported in supplementary materials are based on total cases

Patients were drawn from 774 children and adolescents (*M* age = 14.37 years, SD = 2.47, range 4–17) referred to The Gender Identity Development Service (GIDS) in London over a 5-year period (from January 2012 to December 2016), and for whom the Associated Difficulties form was completed. The majority of these patients were rated as wishing to live in a binary gender opposite to their birth-assigned gender (93%), with a small minority rated as desiring to live as non-binary (7%). Most were White British (75.4%), with a small representation of other ethnic groups. To our knowledge, none of the patients referred had commenced any form of medical transition (including hormone blockers) prior to first being seen at GIDS. There was a considerable amount of data missing on critical variables, as depicted in Fig. 1 (i.e., flowchart outlining exclusion of cases due to missing data on variables of interest). Missing data were due to clinicians failing to complete the associated difficulties form at assessment or clients dropping out prior to assessments being completed.

To be included in the primary analysis, cases required a diagnosis of gender dysphoria, as well as birth-assigned gender and age to be recorded. A negative gender dysphoria diagnosis was recorded for *n* = 155 patients and *n* = 149 patients did not have gender dysphoria status recorded on their files. Reasons for the former were not recorded but likely include clinicians’ failing to complete the form or patient drop-out prior to gender dysphoria diagnosis being made. In addition, at least one transition variable (living in role and/or name change) needed to be recorded. Considerably more data were missing on the living in birth-assigned gender versus living in affirmed gender variable—than the name change variable (i.e., *n* = 69 additional cases) (again, see Fig. 1). A small amount of data were missing on mental health variables (no more than four cases were missing on either mood, anxiety, or suicide attempts). For ease of reporting sample characteristics, only cases for which data on all three mental health indices were recorded were included in the primary analysis or in secondary analysis completed in supplementary materials.

The final sample for analysis of living in birth-assigned gender versus affirmed gender role status consisted of 288 children and adolescents, of whom 72% were birth-assigned female and 73% had undertaken a partial or full social gender transition (208 birth-assigned female; 210 socially transitioned). All patients with living in birth-assigned gender versus affirmed gender role data also had corresponding name change data. The final sample for analysis of name change status consisted of 357 children and adolescents, of whom 71% were birth-assigned female and 60% had changed their name (253 birth-assigned female; 214 name change).

### Procedure and Measures

Upon first contact with the GIDS, patients and their families undertook in-depth psychological assessment of gender dysphoria, comorbid psychiatric disorders, and relevant psychosocial stressors. These assessments involved a minimum of three one-hour assessments with two mental health clinicians (psychiatrists, clinical psychologists, or registered psychotherapists). Assessment sessions involved both child/adolescent patients and their families, assessed individually and together as a family. At the end of the assessment period, the associated difficulties form was completed by both mental health clinicians. Clinicians conferred with one another to ensure agreement on diagnoses. When disagreement in opinion was present—clinicians discussed the matter (or assessed further) until agreement could be made. Referral letters and clinician reports referencing mental health functioning prior to contact with the GIDS were also taken into account in making these judgments (e.g., an autism spectrum disorder [ASD] diagnosis could be gleaned via letters in many instances). The associated difficulties form involved 29 questions relevant to demographics, psychosocial stressors, and DSM-5 diagnoses. These questions are outlined in detail in a past study from our service using this data (Holt et al., 2016). The full list of variables in the Associated Difficulties form is reported in Supplementary Materials. In the present study, we examined data on 10 variables from the Associated Difficulties Form: birth-assigned gender, ethnicity, age at time of referral, age of first gender dysphoria symptoms, social transition (no, partly, yes), name change (no, partly, yes), the presence of current mood or anxiety disorders at time of assessment, and past suicide attempts.

The key variables of social transition, “living in birth-assigned gender versus affirmed gender role” and “name change,” were originally rated in three categories: “no” (i.e., no social transition/name change), “partly” (i.e., partial social transition/name change), or “yes” (i.e., full social transitioned/name change”) (consistent with how social transition was assessed by Steensma et al., [2013]). Because very few respondents were rated as partially socially transitioned or as partially using a new name, this was not a viable cell for analysis. We, therefore, combined partially and fully transitioned patients into a single group, creating dichotomous variables for both living in role and name change (0 = No transition or No name change, 1 = Any social transition or Any name change). This dichotomization is consistent with how these variables were treated by Steensma et al. (2013). See Table 1 for a breakdown of social transition/name change, partial social transition/partial name change, and no social transition/no name change in the gender dysphoria confirmed sample and for total cases. Mood difficulties reflected a full diagnosis of a DSM-5 depressive disorder (e.g., major depressive disorder or persistent depressive disorder). Anxiety difficulties reflected a full diagnosis of a DSM-5 anxiety disorder (e.g., social anxiety disorder, panic disorder, generalized anxiety disorder). Suicide attempts reflected any suicide attempt occurring in the client’s past. All data were anonymized in order to ensure the confidentiality of patients.

**Table 1:** Breakdown of patients “living in role” (yes, partly, no) and “name change” (yes, partly, no) among those with a gender dysphoria diagnosis confirmed as well as total cases (irrespective of gender dysphoria status)

	Gender dysphoria diagnosis confirmed		Total cases	
	Living in role (% [*n*])	Name change (% [*n*])	Living in role (% [*n*])	Name change (% [*n*])
Yes	59.4% (171)	59.9% (214)	50.7% (248)	49.3% (303)
Partly	13.5% (39)	2.2% (8)	12.7% (62)	2.3% (14)
No	27.1% (78)	37.8% (135)	36.6% (179)	48.5% (298)

### Data Analytic Plan

In addition to the variables “living in one’s affirmed gender role” and “name change,” a third social gender transition variable was computed (“social transition composite”) which was the sum of “living in one’s affirmed gender role” and “name change” resulting in a score from 0 (neither “living in one’s affirmed gender role” nor “name change”) to 2 (both “living in one’s affirmed gender role” and “name change”). Age was treated as a continuous variable. Mood and anxiety difficulties were rated as present or absent, as was history of a suicide attempt.

We planned to use binomial logistic regression to examine whether the association between living in role, name change, and a composite of living in role and name change, and dependent variables (mood, anxiety, or suicide attempt) were moderated by age and birth-assigned gender. We examined interactions between age and birth-assigned gender, and the social transition variables as past theoretical and empirical research identify different pathways to gender dysphoria and different comorbidities associated with gender dysphoria, based on age at referral and birth-assigned gender (Aitken et al., 2015; Lawrence, 2010).

For each dependent variable, regression models took the same form. In Step 1, we examined the main effect of “living in role” or “name change,” “birth-assigned gender,” and “age” on the dependent variable of interest. In Step 2 (which was only interpreted if the Chi-square change for Step 2 was significant), we added interactions between Birth-assigned gender × Age, Birth-assigned gender × Living in Role/Name Change/Composite, and Living in Role/Name Change/Composite × Age. In Step 3, we added the three-way interaction between Birth-assigned gender × Age × Living in Role/Name Change/Composite (again, Step 3 was only interpreted if the Chi-square change for Step 3 was significant). Whereas analysis reported here is for those patients who had a positive gender dysphoria diagnosis—we also completed identical analysis on the full sample of referrals to the GIDS (with or without a positive diagnosis of gender dysphoria—see Supplemental Materials. Findings were identical to those completed on the gender dysphoria cohort). The critical α utilized in all analyses was *p* < 0.05.

As our sample size was determined by available data, we wanted to assess the minimum effect sizes that the current sample was equipped to identify with a power of 0.80 (*α* < 0.05). Thus, the existing values of the sample (Social Transition: 179/310 for Living in birth gender/living in affirmed gender, respectively, as well as Social Transition: 312/303; for still using birth name/name already changed, respectively) were inserted to a power calculator (G*Power version 3.1.9.7) for logistic regression to determine the smallest effect sizes that the social transition indicators (living in role and name change) are well powered to detect. The analyses indicated that, given the existing data, the analyses had 80% power to detect an effect size of 1.84/1.70 (in odds ratio terms) for living in role/name change, respectively. That means that given the data, the analyses were well powered to identify even small-medium effect sizes that may differ as a function of the main variable of interest—social transition.

## Results

### Descriptive Statistics

Of those in our sample, 78.4% of birth-assigned females and 58.8% of birth-assigned males had either partially or fully socially transitioned prior to assessment, and 69.2% of birth-assigned females and 37.5% of birth-assigned males had changed their name prior to assessment (see Tables 2 and 3). Chi-square tests (a 2 [Age group] by 2 [Social transition indicators]) demonstrated that, among AFAB patients, proportions of prepubertal (4–12) versus adolescent (13–17) patients who were “living in role” (*χ*^2^(1) = 0.34, *p* = 0.56) and had undergone “name change” (*χ*^2^(1) = 0.10, *p* = 0.75)—were similar (*p* > 0.5’s). On the other hand, among AMAB patients, adolescent AMABs were significantly less likely to be “living in role” (*χ*^2^(1) = 9.55, *p* = 0.002) bthan prepubertal AMABs, but did not differ in likelihood of “name change” compared to prepubertal AMABs (*χ*^2^(1) = 0.09, *p* = 0.77).

**Table 2:** Descriptives for mood disorder, anxiety disorder, and suicide attempt prevalence in those living in their birth gender role versus living in their affirmed gender role

	AFAB		AMAB	
	Living in birth gender (*n* = 45)	Living in affirmed gender (*n* = 163)	Living in birth gender (*n* = 33)	Living in affirmed gender (*n* = 47)
4–12^a^ (years)	25.9% (7)	74.1% (20)	13.6% (3)	86.4% (19)
13–17^a^ (years)	21.0% (38)	79.0% (143)	51.7% (30)	48.3% (28)
Age mean (SD)	14.40 (2.28)	14.61 (1.93)	14.94 (2.52)	12.62 (3.74)
Mood disorder^b^	44.4% (20)	53.4% (87)	63.6% (21)	34.0% (16)
Anxiety disorder^b^	33.3% (15)	33.7% (55)	33.3% (11)	27.7% (13)
Suicide attempt^b^	6.7% (3)	11.7% (19)	6.1% (2)	8.5% (4)

^a^The percentages in this row reflect the percentages of individuals in that specific age group who live in birth vs affirmed gender

^b^The percentages in this row reflect the percentages of individuals with the psychopathological indicator as a function of the gender they are living in

**Table 3:** Descriptives for mood disorder, anxiety disorder, and suicide attempt prevalence in those using their birth name versus affirmed name

	AFAB		AMAB	
	Birth name (*n* = 78)	Name change (*n* = 175)	Birth name (*n* = 65)	Name change (*n* = 39)
4–12^a^ (years)	33.3% (10)	66.7% (20)	60% (15)	40% (10)
13–17^a^ (years)	30.5% (68)	69.5% (155)	63.3% (50)	37.7% (29)
Age mean (SD)	14.55 (1.82)	14.61 (2.03)	14.04 (3.12)	13.56 (3.42)
Mood disorder^b^	48.7% (38)	54.3% (95)	46.2% (30)	46.2% (18)
Anxiety disorder^b^	29.5% (23)	35.4% (62)	33.8% (22)	20.5% (8)
Suicide attempt^b^	9.0% (7)	13.1% (23)	6.2% (4)	10.3% (4)

^a^The percentages in this row reflect the percentages of individuals in that specific age group who live in birth vs. affirmed gender

^b^The percentages in this row reflect the percentages of individuals with the psychopathological indicator as a function of the gender they are living in

Based on clinical assessment and referral documents, among patients with a diagnosis of gender dysphoria and for whom name change and/or living in role status was recorded, 52.6% of birth-assigned females and 46.2% of birth-assigned males were experiencing mood difficulties, 33.6% of birth-assigned females and 28.8% of birth-assigned males were experiencing anxiety and 11.9% of birth-assigned females and 7.4% of birth-assigned males had past suicide attempts. Tables 2 and 3 show these variables based on participant’s birth-assigned gender, age at referral (4–12 years of age, or 13–17 years of age), and living in role/name change status. Chi-square analyses indicated that there were no significant differences (*p*’s > 0.10) in any of the pathological indicators (i.e., mood disorders, anxiety disorders, and suicide attempted), as a function of the social transition indicators (i.e., living in birth/assigned gender, name change), within each of the two groups for either AFAB or AMAB, except in one case (out of the 12 tests). The sole exception indicated that, among AMABs, mood disorder was more common among individuals living in the birth (vs. affirmed) gender (*χ*^2^(1) = 6.83, *p* = 0.009).

### Correlation Matrix: Social Transition, Birth-Assigned Gender, Age, and Mental Health Status

First, we examined zero-order correlations between all variables. As we had more data on patients who had “name change” (Table 4) than data in “living in role” or both (Table 5), we present two tables. Perusal of the correlation matrixes showed that, relative to birth-assigned females, birth-assigned males were younger at referral, less likely to be “living in role,” or to have changed their name. Age at referral was positively associated with the presence of a mood or anxiety disorder and a suicide attempt. As would be expected, all indices of social transition were highly correlated, as were all indices of mental health status. Critically, there was no significant association between “living in role” and “name change” on the mental health variables.

**Table 4:** Correlations between birth-assigned gender, age, name change, and mental health outcomes (*n* = 357)

	*M*	SD	AFAB vs. AMAB	Age	Name change	Mood	Anxiety	Suicide attempt
AFAB vs. AMAB	1.30	.46						
Age	14.06	2.72	− .136					
Name change	1.66	.47	− .198**	− .081				
Mood	1.50	.50	− .058	.272**	.051			
Anxiety	1.34	.48	− .046	.091	.013	.332**		
Suicide attempt	1.90	.30	− .061	.153**	.078	.195**	.073	

Assigned Female at Birth (AFAB) = 1; Assigned Male at Birth (AMAB) = 2; Name Change, Mood, Anxiety, Suicide Attempt (No = 1; Yes = 2)

Note. *p < .05, **p < .01

**Table 5:** Correlations between birth-assigned gender, age group, social transition status, and mental health outcomes (*n* = 288)

	*M*	SD	AFAB vs. AMAB	Age	In role	Name change	Social composite	Mood	Anxiety	Suicide attempt
AFAB vs. AMAB	1.28	.45								
Age	14.29	2.54	− .176**							
In role	1.73	.45	− .198**	− .081						
Name change	1.64	.48	− .314**	.069	.458**					
Social transition composite	3.37	.79	− .302**	− .004	.842**	.866**				
Mood	1.50	.50	− .047	.288**	− .031	.022	− .004			
Anxiety	1.33	.47	− .035	.119*	− .009	− .006	− .009	.355**		
Suicide attempt	1.90	.30	− .047	.171**	.068	.098	.098	.234**	.047	

Assigned Female at Birth (AFAB) = 1; Assigned Male at Birth (AMAB) = 2; In role, Name Change, Social Composite, Mood, Anxiety, Suicide Attempt (No = 1; Yes = 2)

Note. *p < .05, **p < .01

### Living in Role: Logistic Regressions

#### Mood and Anxiety Difficulties and Suicide Attempts

Table 6 shows the results from the binomial logistic regressions assessing whether living in one’s affirmed gender (i.e., having socially transitioned), birth-assigned gender, and age at assessment (and their two-way and three-way interactions) predicted the likelihood of mood and anxiety difficulties or past suicide attempts. For the regression on mood difficulties and suicide attempts, a main effect of age was observed, such that older patients were more likely to report mood issues and past suicide attempts but not anxiety issues. Living in role and birth-assigned gender were not associated with mood, anxiety, or suicide attempts. Likewise, Step 2 and Step 3 were not significant, indicating no two-way or three-way interactions were observed (*p’*s > 0.05).

**Table 6:** Logistic regressions predicting the likelihood of a mood disorder, anxiety disorder, and suicide attempt in AFAB versus AMAB referrals living in their birth-assigned gender role or affirmed gender role (n = 288)

	Mood disorder			Anxiety disorder			Suicide attempt		
Variable	*β*	*p*-value	Exp (*β*)	*β*	*p*-value	Exp (*β*)	*β*	*p*-value	Exp (*β*)
Model 1									
Step *χ*^2^(3)	25.83	< .001		4.40	.221		14.99	.002	
Nagelkerke *R*^2^	.11			0.21			.11		
AGAB	− .02	.942	.98	− .09	.775	.92	− .15	.770	.86
Age	.27	< .001	1.30	.11	.055	1.12	.48	.005	1.62
In role	− .04	.898	.96	− .01	.961	.99	.71	.188	2.04
Model 2									
Step *χ*^2^(7)	32.78	.073		4.51	.991		15.54	.906	
Nagelkerke *R*^2^	.14			.02			.03		
AGAB	− 1.15	.640	.32	− .16	.940	.85	3.18	.568	23.93
Age	.37	.306	1.45	.18	.556	1.19	.81	.386	2.24
In role	4.29	.102	72.61	.67	.761	1.95	.45	.950	1.57
AGAB × Age	.18	.186	1.20	.01	.951	1.01	− .24	.463	.79
AGAB × In role	− .21	.232	.82	− .04	.752	.96	− .00	.998	1.00
Age × In role	− .94	.152	.39	− .03	.967	.97	.20	.863	1.22
Model 3									
Step *χ*^2^(8)	32.86	.769		5.55	.309		15.63		
Nagelkerke *R*^2^	.14			.03			.11		
AGAB	1.84	.856	6.32	7.11	.340	1224.73	− 5.05	.870	.01
Age	.63	.509	1.87	.91	.266	2.48	.12	.965	1.12
In role	6.40	.403	599.29	6.78	.307	874.00	− 5.40	.805	.01
AGAB × Age	− .02	.978	.98	− .48	.330	.62	.29	.883	1.33
AGAB × In role	− .35	.497	.71	− .46	.303	.63	.37	.790	1.45
Age × In role	− 2.56	.637	.08	− 4.12	.312	.02	4.58	.776	97.64
AGAB x Age × In role	.11	.764	6.32	.28	.310	1.32	-.28	.784	.76

AGAB = Assigned Gender at Birth (1 = Female; 2 = Male); In role, Mood, Anxiety, Suicide Attempt (No = 1; Yes = 2)

### Name Change: Logistic Regressions

#### Mood and Anxiety Difficulties and Suicide Attempts

Table 7 reports results from the binomial logistic regressions assessing whether name change, birth-assigned gender, and age (and all interactions between these variables) were related to the likelihood of mood or anxiety issues or suicide attempts. As above, age was positively associated with likelihood of mood issues and suicide attempt, but not anxiety. Name change and birth-assigned gender were not associated with mental health status. No two-way or three-way interactions were significant (*p’*s > 0.05).

**Table 7:** Logistic regressions predicting the likelihood of a mood disorder, anxiety disorder, and suicide attempt in AFAB versus AMAB referrals with their birth-assigned name versus affirmed name (n = 357)

	Mood disorder			Anxiety disorder			Suicide attempt		
Variable	*β*	*p*-value	Exp (*β*)	*β*	*p*-value	Exp (*β*)	*β*	*p*-value	Exp (*β*)
Model 1									
Step *χ*^2^(3)	29.27	< .001		3.53	.317		14.14	.004	
Nagelkerke *R*^2^	.11			0.14			.08		
AGAB	− .07	.781	.93	− .17	.524	.84	− .30	.506	.75
Age	.26	< .001	1.30	.08	.107	1.09	.36	.006	1.43
Name change	.19	.429	1.21	.00	.986	1.00	.47	.233	1.61
Model 2									
Step *χ*^2^(7)	33.64	.224		6.59	.382		14.66	.915	
Nagelkerke *R*^2^	.12			.03			.08		
AGAB	− 3.31	.127	.04	.47	.806	1.60	1.76	.683	5.79
Age	.06	.845	1.06	.03	.930	1.03	.73	.253	2.07
Name change	1.19	.545	3.30	1.34	.481	3.82	1.57	.725	4.83
AGAB × Age	.22	.091	1.25	.05	.682	1.05	− .16	.535	.85
AGAB × Name change	− .02	.968	.98	− .92	.102	.40	.25	.777	1.29
Age × Name change	− .07	.608	.94	− .01	.930	.99	− .09	.739	.91
Model 3									
Step *χ*^2^(8)	33.64	.997		8.04	.227		14.77		
Nagelkerke *R*^2^	.12			.03			.08		
AGAB	− 3.28	.621	.04	7.07	.236	1174.20	− 3.31	.841	.04
Age	.06	.924	1.07	.73	.284	2.08	.27	.864	1.30
Name change	1.22	.830	3.37	7.79	.185	2415.99	-2.56	.848	.08
AGAB × Age	.22	.619	1.25	− .41	.315	.67	.17	.874	1.18
AGAB × Name change	− .04	.992	.96	− 5.28	.168	.01	3.18	.728	23.96
Age × Name change	− .07	.861	.94	− .45	.254	.64	.18	.839	1.19
AGAB × Age × Name change	.00	.997	1.00	.30	.246	1.35	− .19	.747	.83

AGAB = Assigned Gender at Birth (1 = Female; 2 = Male); Name Change, Mood, Anxiety, Suicide Attempt (No = 1; Yes = 2)

### Social Transition Composite: Logistic Regressions

#### Mood and Anxiety Difficulties and Suicide Attempts

Table 8 shows the results from the binomial logistic regressions assessing whether the social transition composite variable, birth-assigned gender, and age (as well as interactions between all variables) predicted the likelihood of mood and anxiety difficulties and suicide attempts. A main effect of age was observed such that older patients had a greater likelihood of mood issues and suicide attempts, but not anxiety issues. The social transition composite variable did not predict mental health status nor did birth-assigned gender. No two-way or three-way interactions were significant (*p’*s > 0.05).

**Table 8:** Logistic regressions predicting the likelihood of a mood disorder, anxiety disorder, and suicide attempt in AFAB versus AMAB referrals based on the social transition composite (n = 357)

	Mood disorder			Anxiety disorder			Suicide attempt		
Variable	*β*	*p*-value	Exp (*β*)	*β*	*p*-value	Exp (*β*)	*β*	*p*-value	Exp (*β*)
Model 1									
Step *χ*^2^(3)	25.81	< .001		4.46	.216		15.86	.001	
Nagelkerke *R*^2^	.11			.21			.11		
AGAB	− .02	.951	.98	− .11	.731	.95	− .03	.949	.97
Age	.27	< .001	1.31	.11	.055	.01	.47	.006	1.60
Social transition	− .01	.949	.99	− .04	.812	.11	.50	.113	1.65
Model 2									
Step *χ*^2^(7)	31.09	.152		4.64	.591		16.53	.881	
Nagelkerke *R*^2^	.14			.02			.12		
AGAB	.39	.572	.25	.41	.849	1.50	4.08	.477	59.08
Age	2.00	.331	1.48	.13	.707	1.14	1.21	.269	3.34
Social transition	.16	.150	7.41	.25	.839	1.28	2.28	.582	9.78
AGAB × Age	− .11	.228	1.18	− .00	.979	1.00	− .25	.431	.78
AGAB × Social transition	− .34	.251	.90	− .01	.936	1.00	− .11	.669	.90
Age × social transition	− 1.37	.359	.71	− .15	.680	.86	4.08	.925	.94
Model 3									
Step *χ*^2^(8)	31.16	.798		7.06	.120		16.65		
Nagelkerke *R*^2^	.14			.03			.12		
AGAB	− 3.96	.706	.02	12.62	.135	303,893.51	− 6.27	.838	.00
Age	.16	.870	1.17	1.39	.140	3.99	.25	.931	1.29
Social transition	1.03	.801	2.79	5.70	.146	297.50	− 1.83	.882	.16
AGAB × Age	.34	.628	1.40	− .83	.141	.43	.40	.834	1.50
AGAB × Social transition	− .04	.886	.96	− .38	.152	.69	.15	.846	1.16
Age × Social transition	.41	.890	1.51	− 3.85	.124	.02	2.78	.736	16.03
AGAB × Age × social transition	− .05	.799	.95	.25	.133	1.29	− .18	.729	.84

AGAB = Assigned Gender at Birth (1 = Female; 2 = Male); Mood, Anxiety, Suicide Attempt (No = 1; Yes = 2)

## Discussion

The present study was among the first to examine whether children and adolescents diagnosed with gender dysphoria who had socially transitioned showed fewer psychological difficulties than those (also with gender dysphoria) who were still living in their birth-assigned gender. Overall, we failed to find robust evidence that social transition (living in one’s affirmed gender role or adopting a name to reflect one’s affirmed gender identity) was associated with mental health status in the short term. Although we found that mood disorders were more common among AMAB who did not transition, in 11 other such comparisons (2—assigned gender at birth × 3—pathological indicators × 2—social transition indicator) there was no indication for differences as a function of social transition. It is possible that the mood finding among AMAB was spurious (e.g., if a Bonferroni correction for multiple tests was used to account for the 12 tests, the AMAB mood difference would have not reached significance). The possibility of the spuriousness is strengthened, as more sensitive analyses that treated age as a continuous rather than as a categorical variable, failed to support that finding.

Our failure to observe significant differences in the mental health status of gender variant children who had socially transitioned versus gender variant children living in their birth-assigned gender is consistent with findings from the methodologically similar studies by Wong et al. (2019) and Sievert et al. (2021). Our findings extend on Wong et al. (who compared published data on the Olson et al. (2016) and Kuvalanka et al. (2017) samples of socially transitioned children with published data on cisgender gender variant children) by failing to find a significant effect of social transition on mental health in a sample of young people all of whom were diagnosed with DSM-5 gender dysphoria—and thus differed in their social transition status—not in their gender dysphoria status. While our findings are consistent with Sievert et al. (2021) in finding social transition was not associated with the mental health status of clinic-referred child patients with a DSM-5 gender dysphoria diagnosis—it extended these findings to adolescents as well. Given adolescent patients comprise the majority of contemporary referrals to gender services (Aitken et al., 2015) and given management of adolescent gender dysphoria has been an area of recent clinical controversy (Littman, 2018; Restar, 2020), the absence of an association between social transition status and mental health status in adolescents is noteworthy.

Notably, contrary to one past study finding a positive association between chosen name use and mental health in gender variant youth (Russell et al., 2018), name change status was not associated with mental health in our sample. The divergence in results could relate to a number of differences between the two studies. First, Russell et al. (2018) measured a more behavioral construct (i.e., in what contexts “are you able to go by your chosen name?”) which, on reflection, seems to assess how safe and affirming one’s social environment is for chosen name use. In our study, by contrast, name change status refers to whether the young person with gender dysphoria had commenced this aspect of their social transition. Second, Russell et al.’s sample was majority young adult (15–21), whereas ours was child and adolescent (4–17). Name change may not be associated with positive mental health outcomes in our sample because: (1) our young people were school age (and schools have been identified as a high-risk environment for harassment of gender variant young people, Martín-Castillo et al. 2020); (2) our sample would have more recently adopted a chosen name (a period when backlash would presumably be higher); and (3) were a clinical sample and thus may have had greater pre-existing mental health vulnerabilities.

As reported in past studies among gender dysphoric cohorts (Holt et al., 2016), we found that the risk of mood difficulties and suicide attempts was higher in gender dysphoric adolescents than gender dysphoric children. Given that psychiatric disorders (including anxiety disorders and depression) and suicidality often first onset in adolescence (e.g., Kessler et al., 2005), this finding is not surprising, and may simply reflect normative developmental processes that make adolescence a vulnerable period for psychopathology in all adolescents. It is also possible, however, that the onset of adolescence in gender dysphoric young people might be a particularly high-risk period (above and beyond that observed in cisgender samples) due to the development of secondary sex characteristics and additional demands related to navigating one’s social environment as a gender diverse person. The failure to find an age-related increase in anxiety disorders in our study was somewhat surprising, given that increased prevalence of anxiety from childhood to adolescence has been reported in non-gender dysphoric samples (Ford et al., 2003). However, developmental literature finds that anxiety is more likely to onset in childhood than is depression or suicidality (Axelson & Birmaher, 2001; Rapee et al., 2009), and thus positive associations between anxiety and age would be expected to be relatively weaker or perhaps nonexistent in some samples.

Supporting previous observation (Holt et al., 2016), prepubertal and adolescent birth-assigned females were more likely to have socially transitioned prior to engagement with specialist gender services than birth-assigned males of equivalent age. One possible explanation for this pattern of findings is that there is less social cost associated with masculine self-presentation among birth-assigned females than feminine self-presentation among birth-assigned males (Shiffman, 2013). It was also notable that almost half of those aged 4–12 were living in their affirmed gender and had changed their name (either partially or fully) prior to contact with the service. In line with observations of Ehrensaft (2016) and recent empirical studies (e.g., Morandini et al., 2022), it appears that an increasing number of parents are facilitating social transition with their gender variant child prior to contact with specialist gender clinics.

### Limitations

There were limitations of the present study that should be kept in mind when interpreting key findings. We did not have sufficient demographic data to determine whether our sample was representative of clinic-referred samples of gender variant youth and therefore how generalizable these findings are. Next, owing to our cross-sectional design, we were not able to capture how benefits (or adversities) related to social transition might unfold overtime. For instance, it is possible that benefits of social transition accrue slowly, perhaps over years, as a young person’s peer and family environment progressively accommodate their affirmed gender. Relatedly, it is possible that social adversity is heightened in the early stages of social transition (e.g., anxiety around passing, misgendering), canceling out benefits of greater gender congruence, with positive effects on mental health only becoming evident as these adversities subside or as coping strategies are developed. Alternatively, young people might experience temporary improvements in mental health related to social transition that subside with time.

The absence of longitudinal data (tracking the same individuals’ mental health status before and after social transition) leaves open the possibility that young people in our sample who socially transitioned were experiencing more severe gender dysphoria than those who had not socially transitioned. As such, it might be the case that social gender transition had ameliorated distress in our socially transitioned children and adolescents but that they failed to demonstrate superior functioning than non-transitioned peers, owing to their more severe presentations at baseline. It is also possible that social transition alone without subsequent medical affirmation (e.g., puberty suppression, gender affirming hormones, or surgery) is insufficient to treat gender dysphoria and that benefits of social transition might occur once young people feel more gender-congruent in their bodies or pass more easily in their affirmed gender following hormone replacement therapy or gender affirming surgeries. Finally, there is a need for caution when interpreting the association (or lack thereof) between likelihood of suicide attempts and social transition status in this study. To the extent that social transition has an ameliorating effect on suicidality, all things being equal, we may expect to see a reduced likelihood of suicide attempts in the group who has undergone social gender transition versus not undergone social transition. However, it is possible that insufficient time has passed for social gender transition to influence suicidality particularly given suicide attempts are rare events. Second, if the socially transitioned group had higher or lower likelihood of suicide attempts prior to their social transition (than the group who did not social transition) this could lead to spuriously attributing this difference to an intervention (social transition) which could not have generated this group difference. Again, longitudinal research is required before any confident claims regarding the relationship between social gender transition and suicide attempts can be made.

Future studies should also measure the moderating influence of transition related discrimination and transition related social support when assessing the mental health consequences of social gender transition. Relevant to this latter possibility, Durwood et al.’s (2021) study, examining parent reports of 265 socially transitioned transgender youth, ages 3–15 (67.2% transgender girls, 32.8% transgender boys) found that parents who reported higher levels of family, peer, and school support for their child’s transgender identity also reported fewer internalizing symptoms in their child. Moreover, peer and school support for the young person’s transgender identity moderated the association between gender-related victimization and internalizing symptoms. Such research will better identify how to avoid or ameliorate negative psychosocial experiences related to social transition.

Our assessment of social gender transition was also limited in a number of respects. Firstly, our sample was almost exclusively binary identified transgender young people (only 7% identified as a non-binary), and thus our assessment of social gender transition was designed with this population in mind. It is unclear what a “full” or “partial” transition looks like for a non-binary individual. In this type of social transition, the goal may be to incorporate some mixture of masculine and feminine presentation or to achieve fluidity in gender presentation across contexts. Future studies should not only aim to examine how social transition may impact well-being in non-binary populations (separated out from binary transgender patients) but also identify more accurate ways of operationalizing and measuring what social gender transition means in non-binary pediatric patients. This is important given that non-binary identities are increasingly prevalent in emerging cohorts of gender variant youngsters (Tollit et al., 2023).

Next, mood and anxiety difficulties were assessed as absent or present based on clinician ratings and relevant referral information. This approach lacks standardization and sensitivity to detect differences in psychological functioning between socially transitioned and non-transitioned patients, which exist in the non-clinical range (Durwood et al., 2017; Olson et al., 2016). Future studies should utilize standardized clinical interviews, as well as standardized self- and parent-report scales, when assessing psychological outcomes of social transition to increase sensitivity to detect differences in mental health if they are present.

### Clinical Implications

We stress the importance of not over interpreting cross-sectional data such as that presented in this study—nor drawing overly simplistic conclusions from our data (e.g., “social gender transition has no benefit”). It is possible that although our socially transitioned patients did not demonstrate superior well-being compared to their non-transitioned counterparts, they were nevertheless functioning better (either in terms of mood/anxiety or gender dysphoria severity, or both) than their own prior functioning pre-social transition. It is also possible that benefits of social transition were not captured in the present study for other reasons; for instance, they might not have had time to emerge or were canceled out by stresses related to adjustment in the short term (even though socially transitioned cohorts may demonstrate superior functioning in the longer term).

So, what may be a take home for parents, clinicians, and educators? Our data suggest that social gender transition may not render immediate and dramatic alleviation of mental health difficulties for all or most children/adolescents suffering with gender dysphoria. If it did, we would expect to have found some lower prevalence of anxiety or depression in our socially transitioned group. Perhaps our study hints that social gender transition alone, at least in the short term, is no panacea to mental health struggles of young people with gender dysphoria and that clinicians and parents should not expect immediate symptom alleviation specific to gender dysphoria or related to mental health more generally. Ongoing contact with mental health services to support young people through social gender transitioned related stressors (e.g., concealment stress, transphobia, misgendering, and adjustment of family, peer, and community to the young person’s gender) is indicated. Clinically we have observed parents of transgender children lament that their child’s distress should have resolved (or resolved more so) now that they have socially transitioned. Although this perspective may be an outlier, we believe it may be present more broadly in clinicians, educators, and parents with only passing familiarity with struggles of young people with gender dysphoria. The present data may encourage relevant parties to set realistic expectations regarding social gender transition (including the need for ongoing parental and clinical care) and encourage a more holistic understanding of the young persons’ struggles that also acknowledges how social gender transition may alleviate one set of challenges (e.g., alleviate felt gender incongruence) while introducing some others (e.g., transphobia, concealment/passing stress)—requiring clinical support of a different type (e.g., assertiveness training). Again, we stress that the present findings do not suggest that social gender transition will not have positive impacts for some children/adolescents in the short term or that it may not have positive effects on well-being in the longer term. We would urge these findings not directly inform treatment without weighing findings from other relevant research, including those demonstrating possible benefits of social gender transition (Durwood et al., 2017; Kuvalanka et al., 2017; Olson et al., 2016).

It would be negligent of us not to acknowledge other interpretations of the null effects of social transition on the mental health of young people in our study. Some might argue that our failure to demonstrate an association between social transition status and mental health outcomes is due to social transition not effectively ameliorating distress in the short, medium, or long-term for a substantial proportion of gender dysphoric children and adolescents. While there was no evidence that social transition had deleterious effects on the mental health of young people in our study, some may argue that in the absence of positive benefits of social transition initiating early social transitions should be approached with caution. Some authors have warned of possible "iatrogenic" effects of early social transition, based on data suggesting childhood social transition is associated with an increased likelihood of persistence of gender dysphoria (Steensma et al., 2011, 2013) into adolescence and adulthood. Given a body of data suggests that the majority of cases of childhood onset gender dysphoria desist before adulthood (Singh et al., 2021; Zucker, 2018, 2020), early social transition may increase the likelihood that gender dysphoria will persist and that hormonal and/or surgical transition will be required to alleviate gender-related distress. It should be stressed that it is beyond the scope of the present study to lend to support to this or other interpretations of the data.

### Conclusion

The present findings, although preliminary, suggest that social gender transition is not associated with mental health status in children and adolescents, at least in the short term. These findings are consistent with the only other study that directly compared clinic-referred youth experiencing gender dysphoria who had socially transitioned with those who had not (Sievert et al., 2021). Critically, longitudinal research is required in order to make more confident claims about the relationship between social gender transition, mental health, gender dysphoria severity, and the broader psychosocial functioning of young people suffering gender dysphoria.

## Supplementary Information

Below is the link to the electronic supplementary material.

Supplementary file1 (DOCX 65 kb)

Supplementary file2 (SAV 65 kb)

## Footnotes

1 In principle, social gender transition may refer to either a binary social gender transition (in which a person changes gender markers and appearance to live in the gender role “opposite” to that assigned at birth) or non-binary gender transition (in which case the person may aim to defy binary gender-roles in their pronouns and appearance in some instances) (Breslow et al., 2021; Matsuno & Budge, 2017). In the present context, we are confining our discussion to binary social gender transition (birth-assigned males socially transitioning to live as girls and vice versa), which to this point is the more studied phenomenon in paediatric gender services and which may be more likely to be associated with a desire for medical transition (at least among adults) (Breslow et al., 2021). We acknowledge the importance of future research examining non-binary social transition in paediatric populations.

2 Some clinically relevant differences can exist between these populations. For instance, “transgender children and adolescents” typically refers to individuals who have socially and perhaps medically transitioned to live as their affirmed gender (e.g., Olson et al., 2016). Cross-gender identified and gender dysphoric children and adolescents, on the other hand, has been used to describe those experiencing clinically significant gender concerns, but who may or may not have socially transitioned to live in their affirmed gender (Wallien & Cohen-Kettenis, 2008). Finally, gender variant, perhaps the loosest grouping, has been used to refer to children and adolescents who exhibit gender non-conforming behaviors and/or identities, which may or may not be causing clinically significant distress or impairment (Riley et al., 2011). Given children and adolescents who are transgender, experience gender dysphoria, and/or are markedly gender non-conforming in behavior or identity, are a heterogeneous group, it can be difficult to generalize findings across samples.
