# Supplementary material for: Is Social Gender Transition Associated with Mental Health Status in Children and Adolescents with Gender Dysphoria?
Source: Arch Sex Behav. 2023 Apr 4;52(3):1045–60. doi: 10.1007/s10508-023-02588-5 (PMC10101898; doi:10.1007/s10508-023-02588-5)
Supplement: Supplementary file 1 — Supplementary file1 (DOCX 65 kb) [file 10508_2023_2588_MOESM1_ESM.docx]

**Supplementary Materials**

**(All cases without Gender Dysphoria Diagnosis verified)**

Table 1. Descriptives for mood disorder, anxiety disorder, suicide attempt prevalence in those living in their birth gender role versus living in their affirmed gender role

|  | AFAB | | AMAB | |
| --- | --- | --- | --- | --- |
|  | Living in Birth Gender (*n* = 93) | Living in Affirmed Gender (*n* = 235) | Living in Birth Gender (*n* = 86) | Living in Affirmed Gender (*n* = 75) |
| 4-12 | 32.7% (18) | 67.3% (37) | 44.6% (25) | 55.4% (31) |
| 13-17 | 27.5% (75) | 72.5% (198) | 58.1% (61) | 41.9% (44) |
| Age (Means/SD) | 14.27 (2.30) | 14.38 (2.35) | 13.36 (3.52) | 12.57 (4.03) |
| Mood Disorder | 48.4% (45) | 51.5% (121) | 59.7% (43) | 40.3% (29) |
| Anxiety Disorder | 35.5% (33) | 36.2% (85) | 30.2% (26) | 26.7% (20) |
| Suicide Attempt | 7.5% (7) | 11.9% (28) | 7.0% (6) | 10.7% (8) |

Table 2. Descriptives for mood disorder, anxiety disorder, suicide attempt prevalence in those using their birth name versus affirmed name

|  | AFAB | | AMAB | |
| --- | --- | --- | --- | --- |
|  | Birth Name (*n* = 159) | Name Change (*n* = 247) | Birth Name (*n* = 153) | Name Change (*n* =56) |
| 4-12 | 51.6% (32) | 48.4% (30) | 73.5% (50) | 26.5% (18) |
| 13-17 | 36.9% (127) | 63.1% (217) | 73.0% (103) | 27.0% (38) |
| Age (Means/SD) | 14.06 (2.44) | 14.59 (2.17) | 13.11 (3.67) | 1309 (3.91) |
| Mood Disorder | 48.4% (77) | 53.3% (133) | 43.1% (66) | 48.2% (27) |
| Anxiety Disorder | 34% (54) | 38.5% (95) | 28.1% (43) | 21.4% (12) |
| Suicide Attempt | 10.1% (16) | 13.0% (32) | 7.2% (11) | 12.5% (7) |

Table 3. Correlations between birth-assigned gender, age, name change, and mental health outcomes (*n* = 615).

|  | M / % | SD | AFAB vs AMAB | Age | Name Change | Mood | Anxiety | Suicide Attempt |
| --- | --- | --- | --- | --- | --- | --- | --- | --- |
| AFAB vs AMAB | 1.34 | .47 |  |  |  |  |  |  |
| Age | 13.95 | 2.92 | -.208** |  |  |  |  |  |
| Name Change | 1.49 | .50 | -.323** | .123** |  |  |  |  |
| Mood | 1.49 | .50 | -.068 | .320** | .070 |  |  |  |
| Anxiety | 1.33 | .47 | -.104** | .178** | .045 | .342** |  |  |
| Suicide Attempt | 1.11 | .31 | -.049 | .143** | .068 | .194** | .079* |  |

Note. Assigned Female at Birth (AFAB) = 1; Assigned Male At Birth (AMAB) = 2; Name Change, Mood, Anxiety, Suicide Attempt (No = 1; Yes = 2).

Table 4. Correlations between natal sex, age group, social transition status, and mental health outcomes (*n =* 489).

|  | M / % | SD | AFAB vs AMAB | Age | In Role | Name Change | Social Composite | Mood | Anxiety | Suicide Attempt |
| --- | --- | --- | --- | --- | --- | --- | --- | --- | --- | --- |
| AFAB vs AMAB | 1.33 | .47 |  |  |  |  |  |  |  |  |
| Age | 13.90 | 2.96 | -.216** |  |  |  |  |  |  |  |
| In Role | 1.63 | .48 | -.244** | .018 |  |  |  |  |  |  |
| Name Change | 1.53 | .50 | -.331** | .146** | .524** |  |  |  |  |  |
| Social Transition Composite | 3.17 | .86 | -.330** | .095* | .868** | .878** |  |  |  |  |
| Mood | 1.49 | .50 | -.055 | .331** | -.007 | .057 | .029 |  |  |  |
| Anxiety | 1.34 | .47 | -.074 | .174** | .009 | .039 | .028 | .366** |  |  |
| Suicide Attempt | 1.10 | .30 | -.031 | .165** | .070 | .080 | .086 | .247** | .080 |  |

Note. Assigned Female at Birth (AFAB) = 1; Assigned Male At Birth (AMAB) = 2; In role, Name Change, Social Composite, Mood, Anxiety, Suicide Attempt (No = 1; Yes = 2).

Table 5. Logistic regressions predicting the likelihood of a mood disorder, anxiety disorder, and suicide attempt in AFAB versus AMAB referrals living in their birth-assigned-gender-role or affirmed-gender-role (n = 489)

|  | Mood Disorder | | | Anxiety Disorder | | | Suicide Attempt | | |
| --- | --- | --- | --- | --- | --- | --- | --- | --- | --- |
| Variable | β | *p*-value | Exp (β) | β | *p*-value | Exp (β) | β | *p*-value | Exp (β) |
| Model 1 |  |  |  |  |  |  |  |  |  |
| Step χ^2^(3) | 58.904 | <.001 |  | 16.972 | <.001 |  | 20.614 | <.001 |  |
| Nagelkerke R^2^ | .151 |  |  | .047 |  |  | .086 |  |  |
| AGAB | .035 | .873 | 1.036 | -.201 | .365 | .818 | .070 | .842 | 1.073 |
| Age | .272 | <.001 | 1.312 | .139 | <.001 | 1.149 | .316 | <.001 | 1.372 |
| In Role | -.061 | .766 | .940 | -.022 | .917 | .978 | .531 | .135 | 1.700 |
| Model 2 |  |  |  |  |  |  |  |  |  |
| Step χ^2^(7) | 65.002 | .107 |  | 17.427 | .929 |  |  |  |  |
| Nagelkerke R^2^ | .166 |  |  | .049 | .896 | .834 | 24.438 | .281 | 1.940 |
| AGAB | -1.397 | .339 | .247 | -.182 | .326 | 1.218 | .102 | .817 | .881 |
| Age | .184 | .392 | 1.202 | .197 | .555 | 2.303 | .663 | .785 | .008 |
| In Role | 1.790 | .225 | 5.990 | .834 | .877 | 1.012 | -.127 | .115 | .945 |
| AGAB x Age | .153 | .079 | 1.165 | .012 | .773 | .879 | -4.796 | .762 | 1.171 |
| AGAB x In Role | -.486 | .280 | .615 | -.129 | .568 | .954 | -.057 | .834 | 1.400 |
| Age x In Role | -.083 | .359 | .920 | -.047 | .896 | .834 | .158 | .077 | 1.940 |
| Model 3 |  |  |  |  |  |  |  |  |  |
| Step χ^2^(8) | 65.274 | .602 |  | 17.569 | .706 |  | 24.444 | .935 |  |
| Nagelkerke R^2^ | .167 |  |  | .049 |  |  | .102 |  |  |
| AGAB | -3.648 | .426 | .026 | -1.673 | .690 | .188 | -.004 | 1.000 | .996 |
| Age | -.046 | .925 | .955 | .034 | .943 | 1.034 | -.196 | .839 | .822 |
| In Role | -.176 | .965 | .839 | -.564 | .886 | .569 | -5.497 | .545 | .004 |
| AGAB x Age | .310 | .325 | 1.363 | .116 | .685 | 1.123 | -.013 | .982 | .987 |
| AGAB x In Role | .872 | .742 | 2.392 | .768 | .751 | 2.155 | .626 | .913 | 1.869 |
| Age x In Role | .054 | .846 | 1.055 | .050 | .854 | 1.051 | .383 | .521 | 1.466 |
| AGAB x Age x In Role | -.095 | .602 | .910 | -.063 | .706 | .939 | -.031 | .935 | .970 |

Note: AGAB = Assigned Gender at Birth (1= Female; 2 = Male); In role, Mood, Anxiety, Suicide Attempt (No = 1; Yes = 2).

Table 6. Logistic regressions predicting the likelihood of a mood disorder, anxiety disorder, and suicide attempt in AFAB versus AMAB referrals with their birth-assigned name versus affirmed name (n = 615)

|  | Mood Disorder | | | Anxiety Disorder | | | Suicide Attempt | | |
| --- | --- | --- | --- | --- | --- | --- | --- | --- | --- |
| Variable | β | *p*-value | Exp (β) | β | *p*-value | Exp (β) | β | *p*-value | Exp (β) |
| Model 1 |  |  |  |  |  |  |  |  |  |
| Step χ^2^(3) | 69.397 | <.001 |  | 24.750 | <.001 |  | 17.789 | <.001 |  |
| Nagelkerke R2 | .142 |  |  | .055 |  |  | .058 |  |  |
| AGAB | .007 | .970 | 1.007 | -.353 | .081 | .703 | -.103 | .742 | .903 |
| Age | .260 | <.001 | 1.297 | .143 | <.001 | 1.154 | .232 | .001 | 1.261 |
| Name Change | .137 | .446 | 1.147 | .000 | 1.000 | 1.000 | .309 | .276 | 1.362 |
| Model 2 |  |  |  |  |  |  |  |  |  |
| Step χ^2^(7) | 74.122 | .534 |  | 27.289 | .468 |  | 20.499 | .439 |  |
| Nagelkerke R2 | .151 |  |  | .060 |  |  | .066 |  |  |
| AGAB | -2.008 | .116 | .134 | .117 | .927 | 1.124 | 1.254 | .576 | 3.505 |
| Age | .164 | .364 | 1.179 | .217 | .247 | 1.242 | .263 | .446 | 1.301 |
| Name Change | .956 | .448 | 2.600 | 1.712 | .186 | 5.537 | -2.286 | .344 | .102 |
| AGAB x Age | .130 | .095 | 1.139 | .018 | .814 | 1.018 | -.140 | .327 | .869 |
| AGAB x Name Change | .110 | .790 | 1.116 | -.546 | .212 | .579 | .518 | .407 | 1.678 |
| Age x Name Change | -.065 | .397 | .937 | -.071 | .359 | .932 | .127 | .382 | 1.136 |
| Model 3 |  |  |  |  |  |  |  |  |  |
| Step χ^2^(8) | 74.227 | .745 |  | 27.961 | .412 |  | 21.157 | .417 |  |
| Nagelkerke R2 | .152 |  |  | .062 |  |  | .068 |  |  |
| AGAB | -.945 | .788 | .389 | 2.796 | .431 | 16.379 | 6.484 | .357 | 654.653 |
| Age | .272 | .474 | 1.313 | .495 | .210 | 1.640 | .807 | .302 | 2.241 |
| Name Change | 1.985 | .562 | 7.276 | 4.420 | .220 | 83.067 | 3.412 | .652 | 30.317 |
| AGAB x Age | .056 | .818 | 1.057 | -.169 | .488 | .845 | -.489 | .293 | .613 |
| AGAB x Name Change | -.621 | .787 | .538 | -2.437 | .311 | .087 | -3.272 | .506 | .038 |
| Age x Name Change | -.137 | .560 | .872 | -.259 | .291 | .772 | -.249 | .614 | .779 |
| AGAB x Age x Name Change | .051 | .746 | 1.053 | .132 | .421 | 1.141 | .251 | .434 | 1.285 |

Note: AGAB = Assigned Gender At Birth (1= Female; 2 = Male); Name Change, Mood, Anxiety, Suicide Attempt (No = 1; Yes = 2).

Table 7. Logistic regressions predicting the likelihood of a mood disorder, anxiety disorder, and suicide attempt in AFAB versus AMAB referrals based on the social transition composite (n = 489)

|  | Mood Disorder | | | Anxiety Disorder | | | Suicide Attempt | | |
| --- | --- | --- | --- | --- | --- | --- | --- | --- | --- |
| Variable | β | *p*-value | Exp (β) | β | *p*-value | Exp (β) | β | *p*-value | Exp (β) |
| Model 1 |  |  |  |  |  |  |  |  |  |
| Step χ^2^(3) | 58.816 | <.001 |  | 16.965 | <.001 |  | 20.899 | <.001 |  |
| Nagelkerke R2 | .151 |  |  | .047 |  |  | .087 |  |  |
| AGAB | .050 | .824 | 1.051 | -.200 | .379 | .819 | .139 | .703 | 1.149 |
| Age | .272 | <.001 | 1.312 | .139 | <.001 | 1.149 | .309 | <.001 | 1.362 |
| Social Transition | -.003 | .982 | .997 | -.008 | .949 | .992 | .324 | .112 | 1.382 |
| Model 2 |  |  |  |  |  |  |  |  |  |
| Step χ^2^(7) | 65.067 | .100 |  | 17.671 | .872 |  | 23.147 | .523 |  |
| Nagelkerke R2 | .167 |  |  | .049 |  |  | .097 |  |  |
| AGAB | -1.536 | .306 | .215 | .123 | .931 | 1.131 | .578 | .845 | 1.782 |
| Age | .272 | .244 | 1.313 | .205 | .352 | 1.228 | -.027 | .958 | .974 |
| Social Transition | 1.199 | .157 | 3.318 | .596 | .466 | 1.815 | -2.036 | .250 | .131 |
| AGAB x Age | .145 | .099 | 1.156 | .010 | .898 | 1.010 | -.063 | .737 | .939 |
| AGAB x Social Transition | -.163 | .536 | .849 | -.163 | .540 | .850 | .168 | .694 | 1.183 |
| Age x Social Transition | -.068 | .197 | .934 | -.027 | .577 | .973 | .140 | .203 | 1.150 |
| Model 3 |  |  |  |  |  |  |  |  |  |
| Step χ^2^(8) | 65.347 | .596 |  | 17.774 | .748 |  | 23.299 | .697 |  |
| Nagelkerke R2 | .167 |  |  | .050 |  |  | .097 |  |  |
| AGAB | -4.080 | .419 | .017 | 1.516 | .740 | 4.552 | 4.454 | .672 | 86.004 |
| Age | .015 | .977 | 1.015 | .358 | .497 | 1.430 | .372 | .750 | 1.450 |
| Social Transition | .057 | .980 | 1.059 | 1.283 | .577 | 3.606 | -.032 | .995 | .968 |
| AGAB x Age | .322 | .354 | 1.380 | -.087 | .782 | .916 | -.320 | .644 | .726 |
| AGAB x Social Transition | .639 | .677 | 1.894 | -.609 | .668 | .544 | -1.172 | .739 | .310 |
| Age x Social Transition | .012 | .940 | 1.012 | -.075 | .634 | .928 | .008 | .982 | 1.008 |
| AGAB x Age x Social Transition | -.056 | .596 | .945 | .031 | .098 | .103 | .088 | .701 | 1.092 |

Note: AGAB = Assigned Gender At Birth (1= Female; 2 = Male); Mood, Anxiety, Suicide Attempt (No = 1; Yes = 2).

Table 8. *Associated difficulties variable list*

| Item # | Item | Values |
| --- | --- | --- |
| 1 | Age | Continuous variable |
| 2 | Ethnicity | unkown  white british  mixed black  other white background  black caribbean  any other mixed background  asian-pakistani  mixed-black carribean and white  asian-indian  asian-chinese  mixed-white and asian  white irish |
| 3 | Age First GD Symptoms | 0-6  7-12  13-18 |
| 4 | Living with | both parents  mother  father  mother and father  mother and stepfather  independently  in foster care  aunt and aunt’s partner  boarding school  adoptive parents  grandparents  alternates between separated parents  supported accommodation  children’s home  girlfriend’s family  adoptive mother  mother and grandparents  unknown |
| 5 | Siblings | .00  1.00  2.00  3.00  4.00  5.00  7.00  8.00  10.00 |
| 6 | Education | yes, mainstream  yes, specialist  no |
| 7 | Birth Assigned Gender | female  male |
| 8 | Sexual Orientation | attracted to females  attracted to males  bisexual  asexual |
| 9 | Living In Role | yes, fulltime  yes, part-time  no |
| 10 | Change Name | yes  no  partly |
| 11 | ASD | yes  no |
| 12 | ADHD | yes  no |
| 13 | Mood | yes  no |
| 14 | Anxiety | yes  no |
| 15 | Psychosis | yes  no |
| 16 | Eating Disorder | yes  no |
| 17 | Bullying | yes  no |
| 18 | Abuse | yes  no |
| 19 | DV | yes  no |
| 20 | Family Break Up | yes  no |
| 21 | Mother Depressed | yes  no |
| 22 | Father Depressed | yes  no |
| 23 | Sibling MH | yes  no |
| 24 | Parents/Alcohol Drugs | yes  no |
| 25 | Self-Harm | yes  no |
| 26 | Suicide Attempt | yes  no |
| 27 | Suicidal Ideation | yes  no |
| 28 | Family Support | yes  no |
| 29 | Desire to live as | male  female  non-binary |

Table 9. Social transition status, demographics, and psychological outcomes among prepubescent children aged 4 – 13 years

|  | Living in gender assigned at birth | | | Partly socially transitioned | | | Fully socially transitioned | | |
| --- | --- | --- | --- | --- | --- | --- | --- | --- | --- |
|  | Male | Female | Total | Male | Female | Total | Male | Female | Total |
| Total  Age | 57.4% (31)  9 (2.65) | 42.6% (23)  10.91 (1.78) | 38% (65)  9.81 (2.49) | 71.4% (15)  8.26 (2.60) | 28.6% (6)  11.67 (2.36) | 12.3% (21)  9.24 (2.83) | 30.8% (20)  9.15 (2.5) | 69.2% (45)  10.62 (2.15) | 32.2% (55)  10.17 (2.49) |
| Age First Symptoms  0 - 6  7 - 12  13 +  *Associated Difficulties* | 58.8% (10)  35.3% (6)  5.9% (1) | 42.1% (8)  34.6% (9)  10.5% (2) | 50% (23)  37% (17)  13% (6) | 64.3% (9)  35.7% (5)  0% (0) | 33.3% (2)  66.7% (4)  0% (0) | 55% (11)  45% (9)  0% (0) | 55.6% (15)  29.6% (8)  14.8% (4) | 45% (18)  32.5% (13)  22.5% (9) | 29.1% (28)  33.3% (19)  17.5% (10) |
| Mood | 16.10% (5) | 17.40% (4) | 16.7% (9) | 13.30% (2) | 33.30% (2) | 19% (4) | 20% (4) | 31.10% (14) | 27.7% (18) |
| Anxiety | 12.9% (4) | 17.4% (4) | 14.8% (8) | 20.0% (3) | 22.2% (2) | 23.8% (5) | 25.0% (5) | 22.2% (10) | 23.1% (15) |
| Suicide Attempt | 3.2% (1) | 4.3% (1) | 3.7% (2) | 6.70% (1) | 0% (0) | 4.8% (1) | 0% (0) | 2.2% (1) | 1.5% (1) |

Table 10. Name change status, demographics, and psychological outcomes among prepubescent children aged 4 – 13 years

|  | No name change | | | Name change | | |
| --- | --- | --- | --- | --- | --- | --- |
|  | Male | Female | Total | Male | Female | Total |
| Total  Age | 68.8% (55)  8.63 (2.55) | 42.2% (38)  10.65 (2.22) | 54.7% (93)  9.46 (2.61) | 31.3% (25)  9.16 (2.76) | 57.8% (52)  11.06 (2.09) | 45.3% (77)  10.44 (2.48) |
| Age First Symptoms  0 - 6  7 - 12  13 +  *Associated Difficulties* | 56% (28)  32% (16)  12% (6) | 39.4% (13)  48.5% (16)  12.1 % (4) | 49.4% (41)  38.6% (32)  12% (10) | 70% (14)  25% (5)  5% (1) | 50% (23)  28.3% (13)  21.7% (10) | 56.1% (37)  27.3% (18)  16.7% (11) |
| Mood | 12.7% (7) | 23.70% (9) | 17.2% (16)^a^ | 24.0% (6) | 34.60% (18) | 31.2% (24)^b^ |
| Anxiety | 14.5% (8) | 13.2% (5)^a^ | 14.0% (13)^a^ | 20.0% (5) | 34.60% (18)^b^ | 29.9% (23)^b^ |
| Suicide Attempt | 3.6% (2) | 1.1% (1) | 3.2% (3) | 4.0% (1) | 3.8% (2) | 3.9% (3) |

Table 11. Social transition status, demographics, and psychological outcomes among prepubescent children aged 14 – 17 years

|  | Living in gender assigned at birth | | | Partly socially transitioned | | | Fully socially transitioned | | |
| --- | --- | --- | --- | --- | --- | --- | --- | --- | --- |
|  | Male | Female | Total | Male | Female | Total | Male | Female | Total |
| Total  Age | 57.1% (56)  15.60 (1.04) | 27.8% (72)  15.36 (1.03) | 35.9% (128)  15.48 (1.03) | 9.2% (9)  16.22 (.67) | 13.1% (34)  15.29 (.97) | 12% (43)  15.50 (.97) | 33.7% (33)  15.82 (.88) | 59% (153)  15.42 (.95) | 52.1% (186)  15.49 (.95) |
| Age First Symptoms  0 - 6  7 - 12  13 +  *Sexual Orientation*  Homosexual  Nonhomosexual | 46.9% (23)  26.5% (13)  26.5% (13)  48% (12)  52% (13) | 46.8% (29)  33.9% (21)  19.4% (12)  58.3% (14)  41.7% (10) | 46.8% (52)  30.6% (34)  22.5% (25)  53.1% (26)  46.9% (23) | 50% (4)  50% (4)  0% (0)  33.3% (1)  66.7% (2) | 51.7% (15)  34.5% (10)  13.8% (4)  50% (7)  50% (7) | 51.4% (19)  37.8% (14)  10.8% (4)  47.1% (8)  52.9% (9) | 50% (14)  25% (7)  25% (7)  23.5% (4)  76.5% (13) | 38.2% (52)  39.7% (54)  22.1% (30)  68.1% (62)  31.9% (29) | 40.2% (66)  37.3% (61)  22.6% (37)  61.1% (66)  38.9% (42) |
| *Associated Difficulties* |  |  |  |  |  |  |  |  |  |
| Mood | 69.9% (39) | 58.3% (42) | 63.3% (81) | 66.7% (6) | 67.6% (23) | 67.4% (29) | 57.6% (19) | 55.6% (85) | 55.9% (104) |
| Anxiety | 39.3% (22) | 43.1% (31)^a,b^ | 41.4% (53) ^a,b^ | 44.4% (4) | 58.8% (20)^b^ | 55.8% (19)^b^ | 30.3% (10) | 35.9% (55)^a^ | 34.9% (65)^a^ |
| Suicide Attempt | 8.9% (5) | 8.3% (6) | 8.6% (11) | 11.1% (1) | 11.8% (4) | 11.6% (5) | 18.8% (6) | 15.1% (23) | 15.8% (29) |

Table 12. Name change status, demographics, and psychological outcomes among prepubescent children aged 14 – 17 years

|  | No name change | | | Name change | | |
| --- | --- | --- | --- | --- | --- | --- |
|  | Male | Female | Total | Male | Female | Total |
| Total  Age | 72.9% (97)  15.54 (.96) | 34.3% (110)  15.22 (1.06) | 45.6% (207)  15.37 (1.02) | 27.1% (36)  15.94 (.83) | 65.7% (211)  15.47 (.47) | 54.5% (247)  15.54 (.93) |
| Age First Symptoms  0 - 6  7 - 12  13 +  *Sexual Orientation*  Homosexual  Nonhomosexual | 43% (37)  32.6% (28)  24.4% (21)  35.3% (12)  64.7% (22) | 50.5% (47)  29% (27)  20.4% (19)  55.3% (21)  44.7% (17) | 46.9% (84)  30.7% (55)  22.3% (40)  45.8% (33)  54.2% (39) | 51.7% (15)  20.7% (6)  27.6% (8)  29.4% (5)  70.6% (12) | 39.6% (72)  41.2% (75)  19.2% (35)  62.9% (66)  37.1% (39) | 41.2% (87)  20.8% (81)  20.4% (43)  36.6% (71)  41.8% (51) |
| *Associated Difficulties* |  |  |  |  |  |  |
| Mood | 60.8% (59) | 55.5% (61) | 58% (120) | 66.7% (24) | 59.7% (126) | 60.6% (151) |
| Anxiety | 36.1% (35) | 39.1% (43) | 37.7% (78) | 27.8% (10) | 41.2% (87) | 39.4% (98) |
| Suicide Attempt | 9.3% (9) | 13.6% (15) | 11.6% (24) | 17.1% (6) | 14.3% (30) | 14.6% (36) |
